# Self‐Assembly of Organic–Inorganic Copolymers Toward Bio‐Inspired Cementitious Composites

**DOI:** 10.1002/advs.77041

**Published:** 2026-08-07

**Authors:** Zonglin Xie, Suning Li, Fuwen Zhong, Gongkun Xiang, Mustapha Jamaa Garba, Yi Tian, Jun Wu, Zhiwu Yu, Qiang Yuan

**Affiliations:** ^1^ School of Civil Engineering Central South University Changsha China; ^2^ National Engineering Research Center for High‐Speed Railway Construction Technology Changsha China; ^3^ China Railway Group Limited Beijing China

**Keywords:** bioinspired materials, cementitious material, disordered collagen‐mineral architecture, organic–inorganic copolymers

## Abstract

Refined by millions of years of rigorous evolution, highly mineralized biological materials have perfected organic–inorganic self‐assembly to break the traditional trade‐off between strength and damage tolerance. Their rigid‐flexible precursor architectures motivate an organic–inorganic copolymerization strategy for the bottom‐up design of cementitious materials, the most pervasive and resource‐intensive engineered products. Herein, as the primary binding phase in cement, calcium silicate hydrate (CSH) is copolymerized with thioctic acid (TA) into a hybrid precursor through a thermodynamically spontaneous process driven by Lewis acid‐base coordination (Ca^2+^‐COO^−^). Hot pressing promotes the synergistic evolution of amorphous CSH into a denser and structurally reorganized framework together with TA crosslinking, resulting in a self‐organized organic–inorganic nanocomposite that resembles the disordered collagen‐mineral architecture in trabecular bone. Notably, the poly(CSH‐TA) composite features a microphase‐homogeneous architecture that delivers an exceptional compressive strength of over 300 MPa at 10% strain. Even more impressively, we demonstrated the intrinsic physical reprocessibility of cementitious materials, allowing retention of over 80% of strength and energy absorption capacity through five consecutive powder‐to‐bulk cycles. The organic–inorganic copolymerization route opens a bioinspired pathway for next‐generation cementitious materials toward achieving multifunctional performance breakthroughs.

## Graphical abstract

Organic–inorganic copolymerization of calcium silicate hydrate and thioctic acid produces a self‐organized nanocomposite inspired by the disordered collagen–mineral architecture of trabecular bone. The resulting material combines a compressive strength above 300 MPa with intrinsic physical reprocessibility, retaining over 80% of its strength and energy absorption capacity after five powder‐to‐bulk cycles.

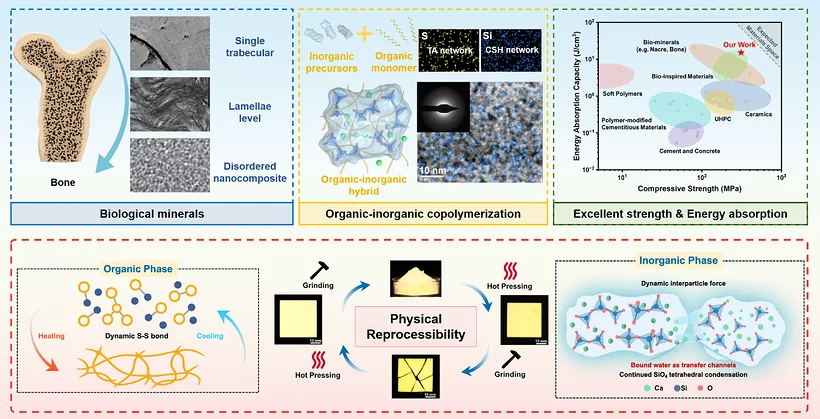

## 1 Introduction

Portland cement, a 200‐year‐old industrial product, remains at the core of global infrastructure. At present, new construction has increasingly addressed pervasive environmental challenges, whereas the existing infrastructure stock faces the pressing need to withstand abnormal events such as earthquakes and tsunamis [1]. Prioritizing high performance of cementitious materials in order to extend service life and reduce maintenance not only lowers the overall project costs but also mitigates life‐cycle CO_2_ emissions, as cement production continues to be the largest single industrial CO_2_ emitter worldwide [2, 3].

Despite their widespread use, traditional cementitious materials consist of heterogeneous, brittle inorganic phases and are characterized by weak interfacial transition zones (ITZs), which fundamentally limit their mechanical properties [4]. Polymer incorporation is among the most widely adopted toughening strategies, and the concept itself is not new, dating back to a patent filed by Cresson in 1923 [5]. Over decades of research and development, polymers for toughening cementitious materials have been broadly classified into two types: film‐forming and reactive. The former mainly include water‐soluble polymers, latexes, and redispersible polymer powders. As water is withdrawn by cement hydration, these admixtures generate continuous films or membranes, binding cement hydrates into a monolithic network. Such a bridging effect enhances tensile‐shear energy dissipation of hardened mortar and concrete [6]. However, film‐forming polymers often retard cement hydration, leading to a substantial reduction in compressive strength. Reactive polymers, such as waterborne resins and monomers, are capable of undergoing crosslinking or in‐situ polymerization in highly alkaline pore solutions, activated by hardeners or initiators [7]. The resulting interpenetrating network has been reported to enhance toughness without sacrificing strength [8].

To advance the technological development of cementitious materials, researchers continue to be fascinated by highly mineralized biological materials that combine remarkable mechanical properties with diverse functionalities [9, 10]. Among various biological materials, nacre attracts attention for its unprecedented combination of high strength and toughness. The characteristic brick‐and‐mortar architecture of nacre comprises ∼95 vol% hard mineral aragonite (CaCO_3_) bonded by layers of organic phase enriched in chitin fibers and proteins. This multiscale rigid‐flexible composite exhibits a toughness that is three orders of magnitude higher than that of mineral CaCO_3_, owing to principal toughening mechanisms such as crack bridging, interlayer sliding, and viscoplastic energy dissipation of the organic phase [11, 12]. Recently, the development of nacre‐like cementitious composites has demonstrated significant breakthroughs in mechanical properties [13]. For example, bioinspired CSH‐based nacre achieves unprecedented flexural strength exceeding 120 MPa and fracture toughness values over 5000 kJ·m^−3^ [14].

Although notable progress has been made, most advances still follow a damage‐for‐toughness paradigm [15, 16]. Such a passive strategy constrains reversibility and undermines the long‐term durability of cement mortar and concrete structures [17]. Even more critically, the inherent anisotropy results in a strength reduction of more than 50% in directions parallel to the platelet orientation [18]. The underlying issue arises from the pronounced physico‐chemical mismatch between flexible organic phases and the brittle cementitious matrix. This incompatibility weakens interfacial cohesion [19] and renders the interface a preferred site for damage initiation as well as a dominant pathway for energy dissipation during crack propagation. Consequently, an emerging design concept is to develop organic–inorganic composites with enhanced structural continuity, even approaching an interface‐free architecture that improves interfacial robustness and elevates the threshold for damage initiation [20].

Natural bone offers another instructive archetype of hierarchical structural materials [21]. Although its remarkable mechanical performance arises from multiscale structural organization, increasing attention has recently been devoted to understanding the contribution of specific nanoscale toughening mechanisms [22]. Within the extracellular matrix, mineralized collagen fibrils serve as the fundamental building blocks, hierarchically organized from the nanoscale to the macroscale. The inorganic phase consists of nanocrystalline carbonated hydroxyapatite (cHA) and amorphous calcium phosphate (ACP), which are dispersed within an organic matrix mainly composed of collagen and non‐collagenous proteins [23]. The integration of the two phases relies on both physical interlocking and strong intermolecular interactions, including hydrogen bonding and partial covalent bonding. Despite sharing similar chemical constituents, bone exhibits two distinct spatial arrangements of organic–inorganic nanocomposites: an ordered phase and a disordered phase. The ordered phase imparts anisotropic strength through aligned fibrillar architectures [24], whereas the disordered phase, characterized by nanoconfined mineral infiltration within the fibrillar network, markedly raises the threshold for damage initiation by regulating load transfer and delaying mineral‐driven local damage (Figure 1a) [25]. Although mimicking the ordered phase has seen notable progress, reproducing the mechanical advantages of the disordered phase remains a major challenge owing to its structural complexity. This challenge has motivated biomineral‐inspired strategies that focus on reproducing the intimate organic–inorganic interactions found in the disordered collagen‐mineral architecture, rather than the complete hierarchy of bone [26, 27]. In such systems, pre‐functionalized organic molecules and inorganic nanoparticles can undergo directed association or selective redistribution under external stimuli, enabling adaptive microstructure evolution and tunable phase organization within the hybrid composite (Figure 1b). For example, Tang et al. [28, 29, 30] pioneered the development of copolymer systems based on organic–inorganic copolymerization around inorganic phase precursors, and they continue to actively advance this research direction, achieving molecular‐level control of interfacial bonding, structural interpenetration, and energy dissipation.

**FIGURE 1:**
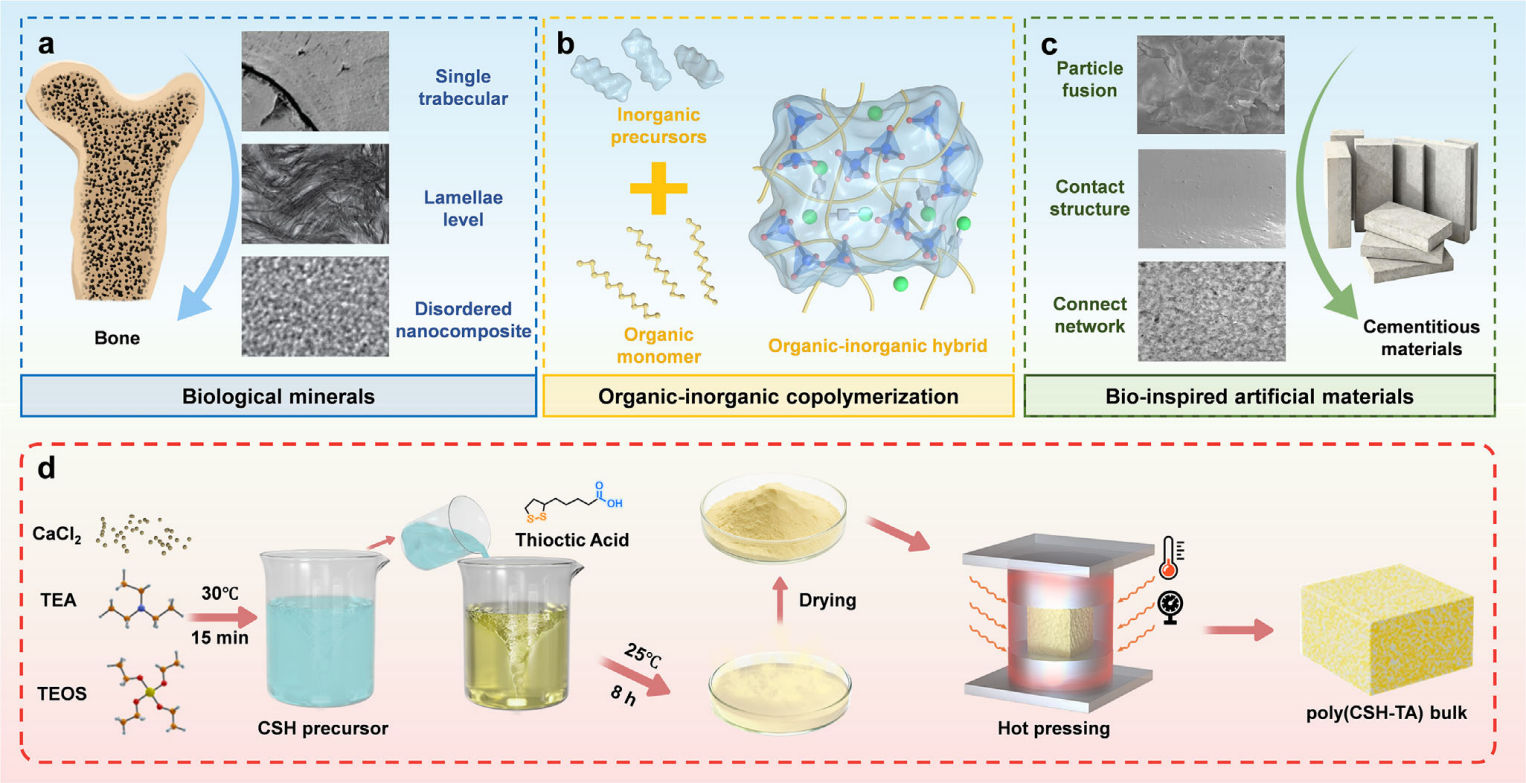
Design concept of cementitious composites inspired by the disordered collagen‐mineral architecture of trabecular bone. (a) Schematic illustration of multiscale toughening mechanisms in bone, (b) conceptual scheme of organic–inorganic copolymerization, (c) the resulting bio‐inspired artificial material, and (d) schematic of the preparation process of bioinspired cementitious composite.

Copolymerization offers valuable guidance for improving the mechanical properties of cementitious materials. Calcium‐silicate‐hydrate (CSH) gel, the primary binding phase during cement hydration, governs the macroscopic concrete performance through its viscoelastic response under load (creep) and volumetric deformation under humidity changes (drying shrinkage) [31]. With a tunable Ca/Si ratio of 1.2–2.3 [32], CSH is a poorly crystalline, defective hydrate phase with partial 14 Å tobermorite‐like features that exhibits glass‐like short‐range order while lacking long‐range periodicity [33, 34], showing certain similarities to ACP in its condensation behavior. On this basis, functionalized organic monomers capable of forming stable interactions with surface‐active sites of CSH under nanoconfinement could be introduced during short‐chain growth (Figure 1c) [35]. When coupled with synergistic crosslinking between the organic and inorganic phases, triggered by external stimuli such as temperature or pressure, this strategy may realize interfacial coupling and structural coevolution. Such an approach provides a promising pathway to overcome the long‐standing interfacial limitations of traditional cementitious composites.

Herein, we demonstrate a practical implementation of the organic–inorganic copolymerization strategy for designing cementitious materials with synergistically enhanced strength and toughness. First, an amorphous CSH precursor and covalent thioctic acid (TA) are copolymerized in an ethanol‐based medium to optimize interfacial compatibility, synthesizing a nanoscale hybrid precursor. Under thermomechanical stimulation, the inorganic CSH segment and organic TA segment generate their respective networks, which are interconnected through CSH‐TA molecular interactions (Figure 1d). This cooperative assembly yields a microphase‐homogeneous composite with a robust and structurally integrated interface. Compared with traditional CSH specimens, poly(CSH‐TA) delivers over a 720% increase in compressive strength and nearly a 35‐times improvement in energy absorption capacity. Furthermore, the poly(CSH‐TA) composite exhibits repeatable powder‐to‐bulk physical reprocessibility.

## 2 Results and Discussion

### 2.1 Synthesis of Nano‐Scale CSH‐TA Copolymer

Synthesized CSH occurred as globular precursors with characteristic sizes of 3–10 nm (Figure 2a). This size range corresponds to an extremely small, experimentally isolable CSH precursor scale obtained by colloidal synthesis, while remaining above the molecular dimension. These nanoparticles are stably dispersed in EtOH as a colloidal sol (Figure S1), providing abundant and compatible surface‐active sites that facilitate interactions with TA molecules. To probe these interactions, we first examined the binding thermodynamics involved in CSH‐TA copolymer formation by performing isothermal titration calorimetry (ITC). As given in Figure 2b and Figure S2, solvent effects were corrected by confirming that the heat associated with TA dilution in EtOH was negligible relative to the CSH‐TA interaction peaks. After this correction was applied, the optimal binding ratio was reached when the TA/CSH molar ratio approached ∼0.3. Further addition of TA caused the calorimetric curve to plateau, signifying near‐complete saturation of the accessible binding sites. Regardless of the titrant concentration, when varying the TA/CSH molar ratio from twofold to fivefold, the equilibrium point remained nearly constant, underscoring that the interaction is an intrinsic characteristic of the CSH‐TA system (Figure S3). In addition, the binding process occurred spontaneously, with a Gibbs free energy (ΔG) of ‐28.61 kJ·mol^−1^ obtained from a single‐site independent model [36]. The strong exothermic enthalpy (ΔH = −74.56 kJ·mol^−1^) stemmed from a sum of all possible intermolecular interactions, and here mainly pointed to the coordination or ionic interaction being likely to participate (Figure 2c), far exceeding typical hydrogen bonds (10–40 kJ·mol^−1^). The unfavorable entropy change (TΔS < 0) originated from both solvation and conformational contributions. During the binding process, the release of weakly bound water in CSH and EtOH molecules surrounding the ‐COOH groups on TA generated positive solvation entropy (ΔS_sol_ > 0). Conversely, TA molecules underwent significant restrictions in conformational freedom (ΔS_conf_ < 0), resulting in a pronounced loss of conformational entropy that outweighed the solvation gain (|ΔS_conf_| > |ΔS_sol_|). Such interfacial ordering, though entropically unfavorable, ultimately enhanced the stability of the CSH‐TA copolymer [37].

**FIGURE 2:**
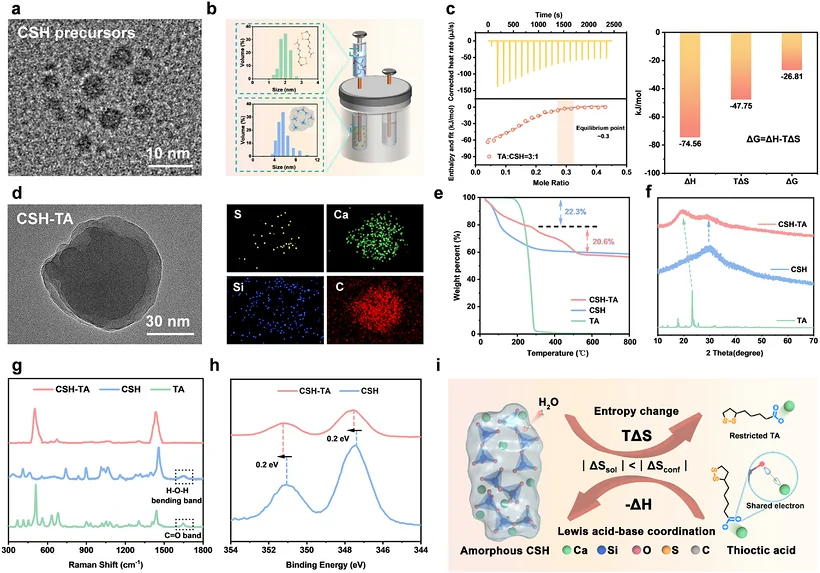
Characterization of physico‐chemical properties of CSH‐TA copolymer. (a) Morphology and particle size of amorphous CSH precursor by TEM, (b) schematic illustration of the ITC measurement and binding thermodynamics between CSH and TA, (c) Gibbs free energy, enthalpy, and entropy changes fitted using a single‐site independent model, (d) TEM‐EDX elemental mapping of the CSH‐TA copolymer in EtOH, (e) TGA curves of CSH, TA, and CSH‐TA copolymer, (f) XRD patterns, (g) Raman spectra, (h) XPS spectra of Ca 2p, (i) Schematic diagram of Lewis acid‐base coordination between Ca sites and ‐COO^−^ groups.

In the liquid phase, the copolymer also maintained a well‐dispersed state with particle sizes of 40–100 nm, as evidenced by TEM, showing no aggregation of the CSH component into foil‐like sheets or clustered structures. Elemental mapping revealed a uniform distribution of S, C, Si, and Ca within individual nanoparticles, demonstrating that TA is chemically incorporated into CSH rather than simply physically mixed (Figure 2d). Within 24 h of drying, the colloidal network gradually transformed from a homogeneous pale‐yellow gel into a xerogel‐like solid that could be readily ground into powder form (Figure S4). The relative contents of the organic and inorganic phases were semi‐quantified by thermogravimetric analysis (TGA), as provided in Figure 2e. The reduced mass loss below 300°C provided additional evidence that copolymerization between TA and CSH led to the partial substitution of chemically bound water within the CSH network. Meanwhile, the thermal decomposition of the TA segment was delayed to a higher temperature range (300°C–500°C), indicating enhanced thermal stability, and was calculated to correspond to an organic content of about 30 wt.%. The residual mass above 500°C remained nearly constant, which represented the inorganic residue. Considering that sulfur from TA was partially retained in the inorganic residue, the Ca: TA molar ratio was calculated by combining the TGA with the XRF (Table S1), yielding the molar ratio of 2.84: 1, consistent with the initial mixing ratio.

XRD revealed that the sharp crystalline peaks of TA disappeared in the CSH‐TA copolymer, indicating that TA no longer retained its original long‐range crystalline state. The pronounced broadening and leftward shift suggested structural rearrangement of the CSH‐based hybrid precursor, likely involving changes in the local layer/interlayer environment of poorly crystalline CSH (Figure 2f). Similar XRD changes in organic‐modified CSH systems have been attributed to organic‐induced disturbance of the CSH framework or interlayer structure [38], which is consistent with the formation of an integrated CSH‐TA hybrid precursor. The interfacial interactions were further probed by Raman spectroscopy and X‐ray photoelectron spectroscopy (XPS). In Raman spectra, the characteristic C═O band at ∼1650 cm^−1^ was substantially weakened in the copolymer, as given in Figure 2g, showing that the ‐COOH groups are no longer free. The reduction of the H─O─H bending band in CSH (∼1600–1650 cm^−1^), together with the negative entropy change (TΔS < 0), revealed a transition from hydroxyl‐based hydrogen bonding to stronger interfacial coupling. Furthermore, XPS of Ca 2p displayed two peaks at ∼347 eV (2p_3/2_) and ∼351 eV (2p_1/2_). Both peaks exhibited a positive shift of ∼0.2 eV in the CSH‐TA copolymer compared to CSH. This shift indicated reduced electron density around Ca, consistent with Lewis acid‐base coordination between Ca sites and ‐COO^−^ groups. In contrast, the Si 2p binding energy remained almost unchanged, indicating that the interaction occurred at Ca─OH sites rather than within the Si‐OH framework of CSH(Figure 2h and Figure S5).

Overall, the above results demonstrated that the CSH‐TA copolymer forms through enthalpy‐dominated coordination between Ca^2+^ and ‐COO^−^ groups, accompanied by interfacial reorganization within the amorphous CSH matrix (Figure 2i). The establishment of compact, ordered interfaces replaced part of the hydrogen‐bonded water network, producing a more cohesive and energetically stable hybrid structure. This Ca^2+^‐COO^−^ coordination imparted strong interfacial cohesion to the copolymer precursor, providing the critical interfacial features required for fabricating high‐performance cementitious composites.

### 2.2 Fabrication of Bio‐Inspired Poly(CSH‐TA) Composites

Hot pressing was employed to induce TA crosslinking as well as regulate the condensation of silicate chains within amorphous CSH. This process required neither water nor ultra‐high temperature sintering. As shown in Figure 3a, particle‐particle fusion gradually occurred, accompanied by the disappearance of distinct boundaries. At 1 min, loosely packed CSH‐TA particles were still visible with clear interfaces. After 10 min of hot pressing, the individual particles had fully coalesced into a continuous bulk structure. The resulting poly(CSH‐TA) exhibited a smooth and compact surface, devoid of discernible micropores. BSEM images revealed a homogeneous and continuous structure extending from the macroscopic to the microscopic scale (Figure 3b). Both the hot‐pressing plane and the perpendicular cross‐section displayed uniform morphology, without cracks, layered textures, or interfacial transition zones (ITZs). At the nanoscale, HAADF‐STEM images (Figure 3c and Figure S6) distinguished the organic and inorganic domains by Z‐contrast: brighter regions correspond to Si‐rich CSH, whereas darker regions correspond to S‐rich TA. These two phases interpenetrated to form a nanoscale network that restricts further condensation of CSH and excessive crosslinking of TA. At the micrometer scale, no obvious phase separation or interfacial discontinuities were observed, indicating a structurally continuous and homogeneous hybrid matrix. In fact, poly(CSH‐TA) exhibited a pore structure markedly different from that of conventional cementitious materials, as given in Figure 3d. Only large gel pores (3–10 nm) and intrinsic small gel pores (∼1.4 nm) associated with amorphous CSH were detected, while capillary pores were absent [39]. This confirmed the development of a highly compact, mesoporous architecture.

**FIGURE 3:**
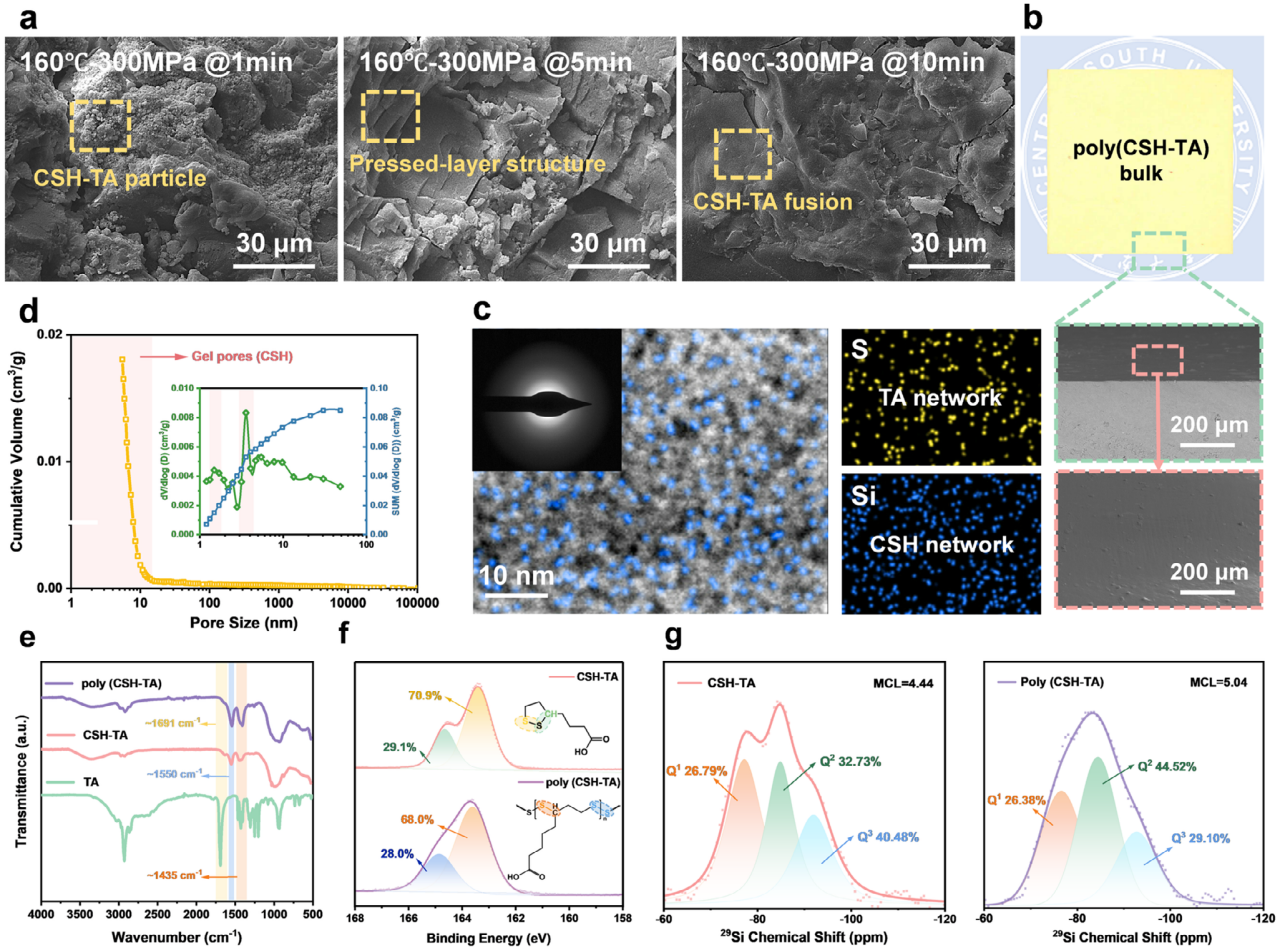
Characterization for physico‐chemical characterization of poly(CSH‐TA) composite. (a) SEM photography of the CSH‐TA powder at different hot pressing stages, (b) digital photograph of poly(CSH‐TA) and its smooth and contact surface structure by SEM, (c) HAADF‐STEM image and EDS elemental mapping of poly(CSH‐TA). The brighter regions correspond to Si‐rich CSH, and the darker regions correspond to S‐rich TA. (d) The pore size distribution, (e) FT‐IR, (f) XPS spectra of S 2p, (g) ^29^Si MAS NMR spectra obtained on CSH‐TA and poly(CSH‐TA).

The chemical‐state evolution of the organic component was identified by Fourier Transform Infrared Spectroscopy (FT‐IR) and XPS. Clear evidence of ‐COOH deprotonation was observed during the copolymerization process. In addition to the residual free ‐COOH band remaining at ∼1691 cm^−1^, two additional features confirmed the formation of carboxylate species. A strong band appeared at ∼1550 cm^−1^, corresponding to the asymmetric stretching vibration of ‐COO^−^, and a pronounced band at ∼1435 cm^−1^ was assigned to its symmetric stretching mode. Hot‐pressing further promoted deprotonation behavior by driving nearly all ‐COOH groups to become involved in the ‐COO^−^ groups, enhancing the interfacial coupling between the two interpenetrating networks (Figure 3e). However, since the FT‐IR signals in the low‐frequency region were weak and overlapped, making it difficult to determine the S element reaction, XPS analysis was performed to further examine the chemical states. The S 2p spectrum revealed a decrease in the C─S component at ∼163.4 eV and an increase in the S‐S component at ∼164.6 eV [40] after hot pressing (Figure 3f). Both peaks shifted toward higher binding energies, reflecting the transformation of sulfur atoms from a shielded cyclic environment to a more electron‐deficient linear configuration. The absence of oxidized sulfur species (>166 eV) indicated that the reaction proceeded via thermally induced disulfide exchange rather than oxidation. Notably, the abundant S─S bonds acted as dynamic covalent linkages that could reversibly break and reform, generating a crosslinked thioether‐disulfide network that enhanced cohesion and enabled reversible chain rearrangement.

Under combined heat and pressure, weakly bonded water molecules escaped and created transient pathways that disrupted the hydrogen‐bond network, thereby enabling the condensation of Si‐OH groups into bridging Si─O─Si linkages [41]. Indeed, confined water in CSH is an active structural component. It dissociates into Si‐OH and Ca‐OH groups and regulates the local Ca^2+^ environment [42, 43]. Such dehydration‐assisted condensation then formed a denser and structurally reorganized CSH framework. Complementary solid‐state ^29^Si MAS NMR resolved the different kinds of silicate tetrahedra. Notably, the new emergence of branching units (Q^3^) at ∼92 ppm in the CSH‐TA copolymer originated from organic‐assisted partial chain branching of the pure CSH network [44] (Figure 3g and Figure S7). Under pressure, the relative fraction of Q^3^ decreases from 40.48% to 29.10%, accompanied by a marked increase in Q^2^ from 32.73% to 44.52%, yet no Q^4^ appeared. Correspondingly, the slight downfield shift of Q^2^ and the upfield relaxation of Q^3^ suggest changes in the local silicate environments, with pressure favoring the stabilization of more extended linear silicate chains. In detail, the mean chain length (MCL) increased from 4.44 to 5.04, demonstrating a moderate enhancement in silicate connectivity. Figure S8 provides additional insight into CSH evolution. With increasing pressing pressure, the Si‐O stretching envelope in the FTIR spectra became broadened, and deconvolution of the 900–1100 cm^−1^ region revealed a higher‐wavenumber Q^3^‐related shoulder shifting from 998 to 1008 cm^−1^. These changes are consistent with pressure‐induced redistribution of local silicate environments and local structural reorganization of amorphous CSH during hot pressing. This evolution was facilitated by the confinement effect of the TA network, which is conceptually analogous to the nanoscale mineral confinement in mineralized collagen [45, 46]. In the present TA/CSH system, such confinement restricted hydrated species mobility and promoted Si─O─Si bridging reactions within the amorphous CSH phase.

Multiscale evidence showed that hot pressing triggered the formation of interconnected CSH and TA networks. Within this interpenetrated architecture, thermally driven disulfide exchange formed a flexible TA framework, while pressure induced short‐range rearrangement of amorphous CSH toward a more connected silicate network. The confined environment allowed the two phases to coevolve synergistically: the TA network moderated CSH mobility and prevented uncontrolled aggregation, whereas the CSH matrix restricted excessive TA crosslinking. Analogous to the disordered phase in bone nanocomposite [25], the crosslinked TA framework imposed nanoscale confinement and internal stress on the CSH phase, guiding its structural organization and raising the crack‐initiation threshold without sacrificing interfacial integrity.

### 2.3 Mechanical Properties of Poly(CSH‐TA) Composites

Three types of specimens, including CSH, poly(CSH+TA) (physical mixture), and poly(CSH‐TA) composite, were evaluated for mechanical properties. In Figure 4a and Figure S9, the poly(CSH‐TA) composite achieved a compressive strength of over 300 MPa and a failure strain exceeding 10%. These values represented a 720% and 440% increase in strength and strain, respectively, compared with pure CSH. In contrast, the physically mixed poly(CSH+TA) specimens showed only limited improvement in strain and even exhibited a reduction in compressive strength, owing to the weak inorganic‐organic interactions and the disconnected microstructural network. Therefore, the mechanical properties of poly(CSH‐TA) demonstrated remarkably synergistic strength and energy absorption capacity of 15.1 J·cm^−3^, exceeding those of a broad spectrum of artificial engineering materials, including cementitious materials, ceramics, and soft polymers (Figure 4b and Table S2). Moreover, the poly(CSH‐TA) composite reached a flexural strength of 40 MPa with a flexural displacement of 4.1% (Figure 4c). It was worth noting that the composite still displayed a brittle fracture mode, and the corresponding flexural toughness was calculated as 0.48 J·cm^−3^, representing a 12‐fold enhancement over pure CSH, yet remaining lower than the values reported for “brick‐and‐mortar” cementitious materials. This difference reflects the distinct toughening mechanisms involved. The high calculated toughness of brick‐and‐mortar or nacre‐like architectures is largely derived from extended post‐peak dissipation after crack deflection and multiple cracking, which can contribute more than 60% of the total energy dissipation. In contrast, the present bio‐inspired copolymerization strategy strengthened intrinsic crack‐initiation resistance through nano‐stitched organic–inorganic interfaces while maintaining near‐ideal isotropy.

**FIGURE 4:**
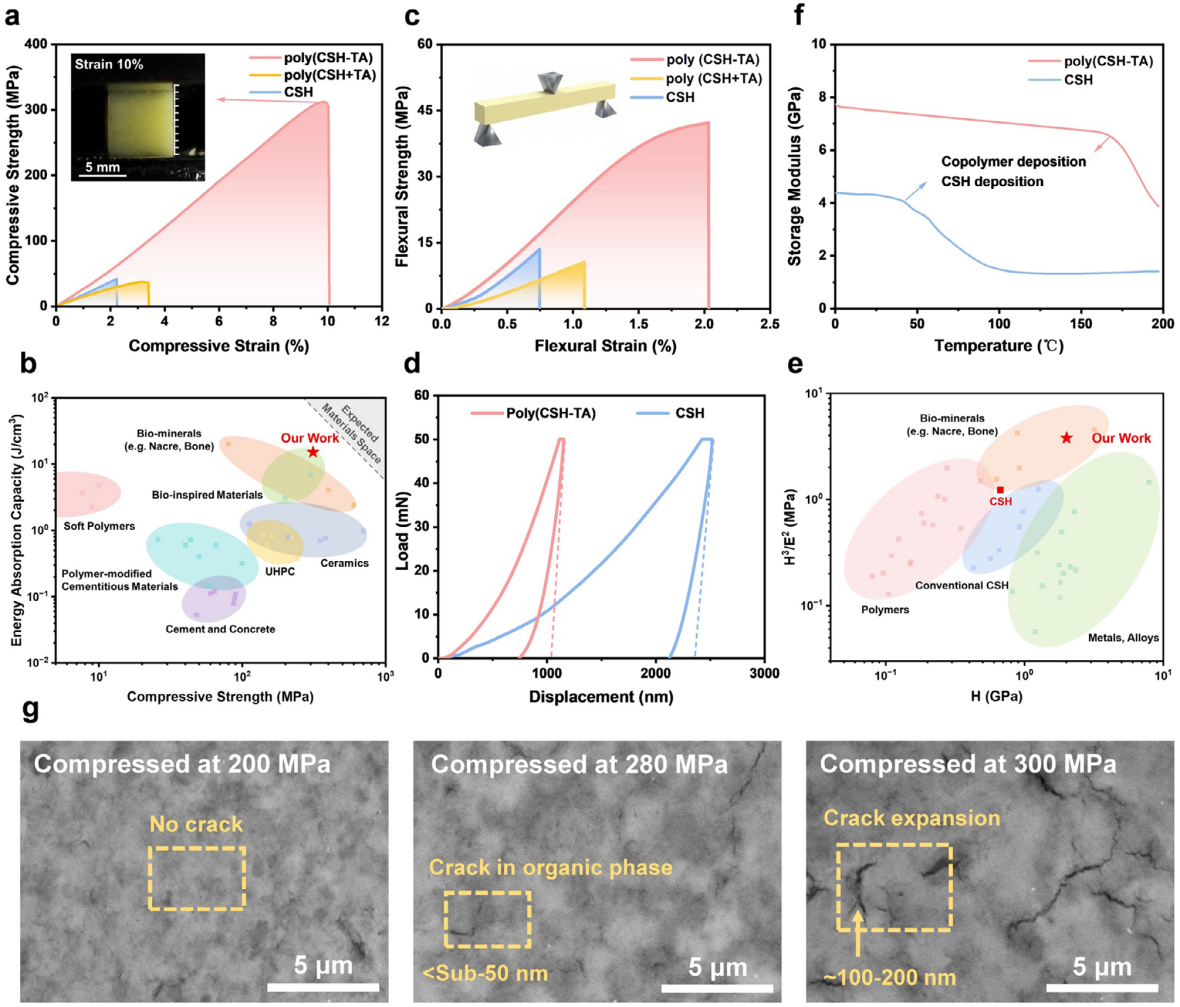
Exceptional mechanical properties of poly(CSH‐TA) composite. (a) Compressive stress‐strain curves of poly CSH, poly(CSH+TA) (physical mixture), and poly(CSH‐TA) composite, (b) comparison of compressive strength and energy absorption capacity among poly(CSH‐TA), bio‐minerals and other typical engineering materials, (c) flexural stress–strain curves, (d) nanoindentation measurements, (e) comparison of H^3^/E^2^ and hardness, (f) storage modulus determined by DMA, (g) BSEM images of poly(CSH‐TA) composite under 200, 280, and 300 MPa, showing the crack initiation and propagation.

Nanoindentation measurements corroborated the above mechanism (Figure 4d). The CSH sample exhibited a hardness of 0.67 GPa and an elastic modulus of 15.68 GPa, whereas the poly(CSH‐TA) composite reached 2.01 and 46.32 GPa, respectively. In the nanocomposite, mechanical loading was carried by a cohesive, integrated network rather than discrete interfaces. Its hardness and modulus not only far exceeded those of conventional cementitious materials but also fell within the range of natural minerals [47] (Figure 4e and Table S3). The temperature dependence of viscoelasticity was also investigated using dynamic mechanical analysis (DMA). As shown in Figure 4f, the storage modulus of the poly(CSH‐TA) composite increased from 4.3 to 7.7 GPa, indicating enhanced stiffness of the composite. In addition, pure CSH showed a rapid loss of storage modulus above 50°C, owing to the thermally induced removal of chemically bound water and the consequent disruption of its hydrogen‐bonded gel network [48]. In contrast, poly(CSH‐TA) maintained its storage modulus with only minimal reduction up to 160°C, underscoring superior thermal stability relative to widely used industrial resins such as commercial polyurethane and epoxy systems [49]. This retention of stiffness was attributed to the continuous inorganic CSH framework with fine mesopores and strong Lewis acid‐base interactions between CSH and TA, which effectively maintained interfacial cohesion and structural integrity under elevated temperatures.

Figure 4g presents the evolution of crack initiation and propagation under compression. At stresses in the elastic deformation regime below 200 MPa, the microstructure remained fully intact, and no discernible cracks were detected. When the load approached ∼280 MPa, nanoscale cracks (<50 nm) appeared. Notably, these early cracks were first observed in TA‐rich nanoscale domains rather than along the organic–inorganic interfaces, suggesting that local damage was preferentially accommodated by the more compliant organic‐rich regions. The absence of interfacial debonding indicates that the structurally continuous organic–inorganic network effectively maintained structural integrity under high compressive stress.

We further evaluated the mechanical response of the poly(CSH‐TA) composite as a function of pressing pressure and temperature, as given in Figure S10. Notably, usable products could be obtained even under mild industrial conditions. A pressure as low as 10 MPa was sufficient to fabricate the specimens, although the particle boundaries remained partially discernible. As the pressure increased up to 500 MPa, the compressive strength of poly(CSH‐TA) composite consistently increased, with strength gains of more than 50% across the tested range. In contrast, the effect of temperature became minimal once the TA ring‐opening threshold was reached. Pressing at room temperature produced a visibly heterogeneous interfacial structure, but after the activation of TA ring‐opening (>120°C), further increasing the temperature up to 160°C led to marginal improvement in mechanical performance.

### 2.4 Recyclability of Poly(CSH‐TA) Composites

With the growing emphasis on green and sustainable construction, the recyclability of cement‐based materials has become increasingly important. Yet conventional concrete is inherently difficult to recycle because hydration produces a permanent and chemically bound microstructure. After hydration has reached an advanced stage, the resulting hydrate network cannot be reverted under standard conditions, and any attempt to do so generally demands high energy usage [50]. Consequently, waste concrete is typically down‐cycled into low‐value recycled aggregates, leading to substantial resource loss [51]. Interestingly, the poly(CSH‐TA) composite was fabricated through a hot‐pressing process. This distinction endowed the material with an intrinsic potential for recyclability, as given in Figure 5a. First, the inorganic framework can be reorganized through dynamic interparticle forces and water‐mediated transport pathways that enable the continuous condensation of silicate tetrahedra under mechanical activation [52]. A further advantage was that the organic component TA offers intrinsic chemical recyclability via reversible disulfide exchange, a dynamic chemistry widely leveraged in sustainable polymer and adhesive systems. Taken together, poly(CSH‐TA) enabled two complementary recycling routes through physical reprocessibility and chemically assisted recycling, providing a foundation for closed‐loop reuse.

**FIGURE 5:**
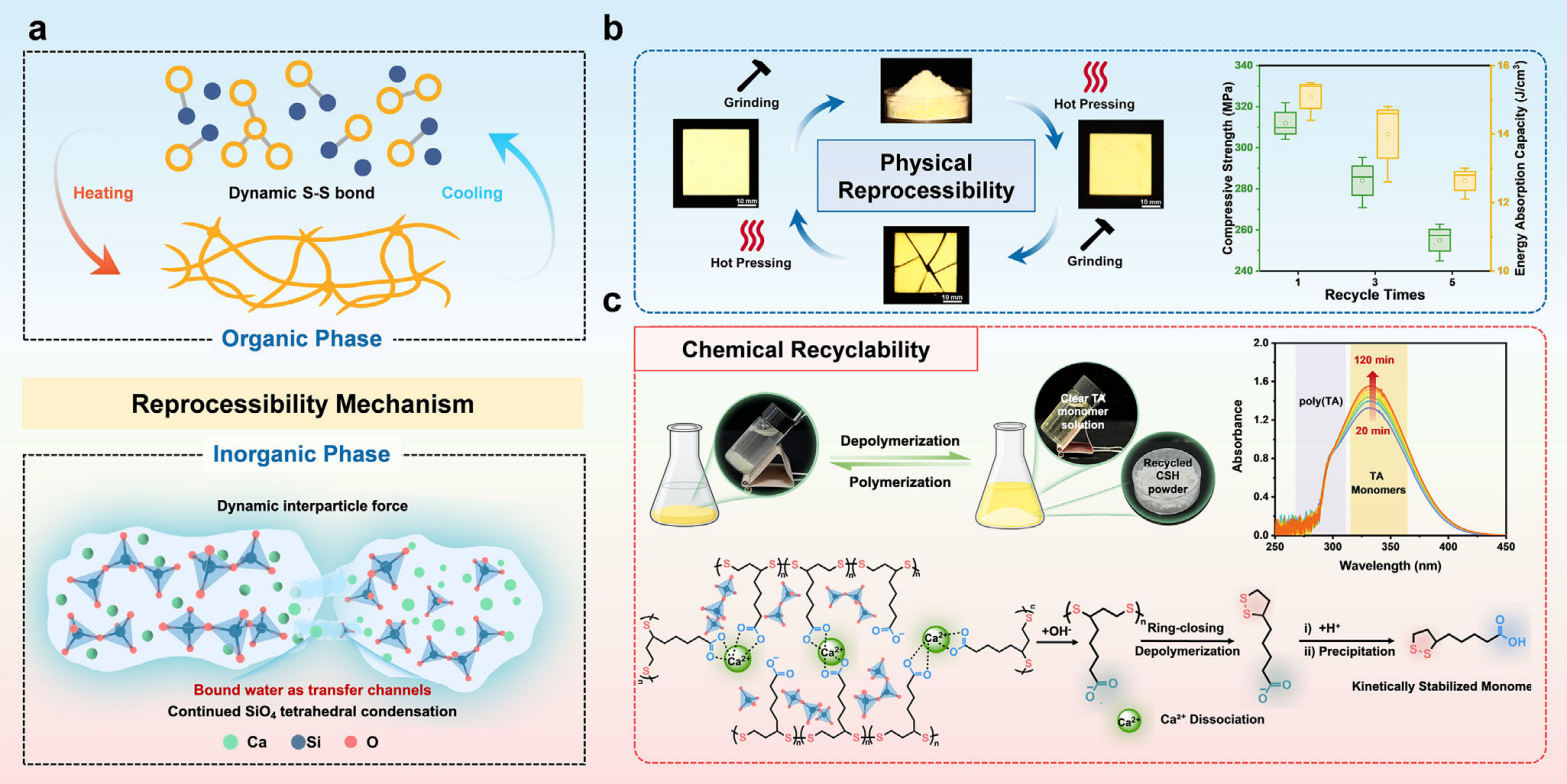
Recyclability of poly(CSH‐TA) composite. (a) Reprocessibility mechanisms of the organic and inorganic phases of poly(CSH‐TA) composite, (b) the closed‐loop physical recycling mode and comparison of mechanical properties before and after multiple recycling, (c) chemical recycling pathway and mechanism, and in situ UV‐vis spectra of the depolymerized poly(Ca‐TA) network.

In physical reprocessibility, we found that fractured fragments of poly(CSH‐TA) composite were readily reprocessed into new specimens under the same hot‐pressing conditions. The recycled materials retained strong mechanical performance even after five reprocessing cycles, with reductions in mechanical properties limited to less than 20%. Notably, the compressive strength remained above 250 MPa, and the energy absorption capacity exceeded 12.6 J·cm^−3^ (Figure 5b). The excellent recyclability was primarily enabled by the reversible disulfide chemistry of the TA network, together with the rebonding of amorphous CSH during hot pressing. Upon heating, the organic phase became malleable due to disulfide bond exchange, which enabled reshaping and consolidation. Upon cooling, these dynamic linkages reformed and restored network integrity. Meanwhile, the amorphous CSH phase remained metastable. After fracture and pulverization, the amorphous CSH could partially re‐establish load‐bearing interparticle contacts under pressure, thereby contributing to structural reversibility [53]. However, repeated hot pressing progressively depletes the physically adsorbed and weakly bound water available for forming these transport pathways, which leads to a slight decline in mechanical performance after repeated recycling (Figure S11).

In chemical recycling, the recovery of the TA segments from the CSH‐TA copolymer required an appropriate solvent. Water or common salt solutions (i.e., NaCl, Na_2_SO_4_) were ineffective because the organic component was not physically adsorbed but was chemically integrated into the CSH matrix through strong Ca^2+^‐COO^−^ coordination (Figures S12 and S13). This coordinated structure replaced part of the hydrogen‐bonded water network and became energetically stabilized, which helped maintain the organic–inorganic coupling of poly(CSH‐TA) powder during immersion in highly concentrated salt solutions. The depolymerization and copolymer dissociation of the TA could be completed within 6 h in a 1 mol·L^−1^ NaOH solution. In situ UV‐vis monitoring further confirmed this process, as a characteristic absorption peak at 330 nm increased progressively with reaction time, as plotted in Figure 5c. After depolymerization, the aqueous NaOH solution containing deprotonated TA was first separated from the solid CSH residue by filtration. The filtrate was then acidified to a pH of about ∼3 to protonate TA and induce its precipitation as yellow solids, which could be collected by filtration and dried [54] (Figure S14). This approach is environmentally friendly, offering an industrial pathway toward sustainable cementitious materials.

## 3 Conclusions

This study established a bio‐inspired intrinsic toughening strategy for cementitious materials and introduced a novel pathway for mesoscopic organic–inorganic integration. By integrating organic–inorganic copolymerization into the cementitious matrix for the first time, a bottom‐up framework was developed that simultaneously enhances mechanical performance while offering promising avenues for functional material design. Based on the above discussion, the following conclusions could be drawn:

1. By tuning the solvent environment, the synthesized CSH precursors (3–10 nm) with abundant reactive surface sites were capable of binding TA. The ensuing organic–inorganic copolymerization was thermodynamically spontaneous and governed by strong exothermic coordination between Ca^2+^ and ‐COO^−^ groups. This enthalpy‐driven interaction displaced part of the hydrogen‐bonded water network and reorganized the amorphous CSH interface, producing hybrid nanoparticles below 50 nm. This resulting structure was energetically stabilized and exhibited enhanced interfacial cohesion.
2. Hot pressing induced TA ring‐opening crosslinking while regulating silicate‐chain condensation in amorphous CSH, enabling the formation of poly(CSH‐TA) with a continuous, pore‐refined architecture extending from the macroscopic to the nanoscale. The resulting material developed an interpenetrating dual‐continuous organic–inorganic network with nanoscale interfacial stitching, which suppressed particle‐boundary defects and eliminated capillary pores. This architecture sustained a compressive strength of over 300 MPa at 10% strain and achieved intrinsic enhancement of both strength and toughness, rather than relying on damage‐mediated toughening strategies. Consequently, poly(CSH‐TA) achieved an energy absorption capacity of 15.1 J·cm^−3^, outperforming existing cementitious materials and approaching the range of selected biological mineralized materials.
3. The intrinsic physical reprocessibility of cementitious materials was demonstrated here as a previously unreported property. This capability arose from the dynamic reversibility of S─S bonds combined with the partial reformation of pressure‐assisted interparticle contact bonding within amorphous CSH. The fractured composites retained over 250 MPa in compressive strength and 12.6 J·cm^−3^ energy absorption capacity even after five powder‐to‐bulk recycling cycles. In addition, the TA component could be depolymerized and recovered under controllable alkaline treatment within the hybrid network. Together, this work offered a new technological pathway for resource recovery and reuse in cementitious materials.
4. This study is particularly relevant to cementitious systems requiring high load‐bearing capacity and damage resistance. At the current stage, the poly(CSH‐TA) composite is more suitable for prefabricated or specialized components than for conventional cast‐in‐place construction because of the relatively high processing temperature and pressure. The proposed organic–inorganic copolymerization strategy also provides a nanoscale design concept that can be integrated with hierarchical architectures, controlled alignment, or fiber reinforcement to further improve mechanical performance while reducing processing requirements.

## 4 Materials and Methods

### 4.1 Materials

Anhydrous calcium chloride (CaCl_2_, CAS 10043‐52‐4), thioctic acid (TA, α‐lipoic acid), and tetraethyl orthosilicate (TEOS) were purchased from Shanghai Macklin Biochemical Co., Ltd. (Shanghai, China). Anhydrous ethanol and triethylamine (TEA) were obtained from Sinopharm Chemical Reagent Co., Ltd. (China). All reagents were used as received without further purification. Deionized water was used throughout all experiments.

### 4.2 Synthesis of CSH‐TA Copolymer

To obtain a homogeneous microscale structure, the copolymer was designed to form at the nanoscale. Amorphous CSH with abundant interfacial binding sites was first synthesized via the sol‐gel method using CaCl_2_ as the calcium source and TEOS as the silica source. To suppress aggregation of CSH particles, TEA was employed as a capping agent. CSH precursors were synthesized as follows: 3.2 g of anhydrous CaCl_2_ was dissolved in 100 mL of ethanol, then 7.6 mL of TEA was added. Next, 7.5 g of TEOS was introduced into the mixture, followed by the dropwise addition of 5 mL of deionized water to initiate TEOS hydrolysis. The resulting solution was stirred at 30°C for 15 min to form a CSH sol.

To match the nanoscale of CSH precursors, the small organic molecule TA was selected for organic–inorganic copolymerization. The CSH‐TA copolymer was synthesized as follows: Before hybrid sol formation, TA was introduced into ethanol (1.07 g in 100 mL), resulting in a 50 mmol·L^−1^ organic solution. 50 mL of the freshly prepared CSH sol was diluted twofold, then added dropwise over 30 min into the TA solution under stirring at 25°C. The reaction was continued for 8 h to obtain the CSH‐TA copolymer sol. Upon completion, the sol was vacuum‐filtered. The pale‐yellow gel retained on the filtration membrane was transferred to a vacuum desiccator and allowed to dry at room temperature for 24 h, and then ground to obtain light‐yellow CSH‐TA powder.

### 4.3 Fabrication of Bulk Sample

Bulk poly(CSH‐TA) samples were fabricated through thermally induced crosslinking of the TA component and pressure‐assisted consolidation of the CSH‐TA hybrid precursor. The dried CSH‐TA powder was loaded into a hot‐pressing mould with a square cavity (side length 40 mm) and compressed using a uniaxial hydraulic press (100‐ton capacity) at 160°C under a uniaxial pressure of 500 MPa for 10 min. After cooling to room temperature, the load was released, and the bulks were demoulded, yielding dense yellow poly(CSH‐TA) bulks. Reprocessing followed the same hot‐pressing protocol. Fractured pieces of the poly(CSH‐TA) bulk were ground into light‐yellow powders. The powder was then placed back into the mould and hot‐pressed under the same temperature and pressure. For comparison, hot‐pressed CSH bulk and poly(CSH+TA) bulk were prepared. Freshly obtained CSH sol was vacuum‐filtered; the white, translucent gel retained on the membrane was transferred to a vacuum desiccator, dried at room temperature for 2 days, and ground to yield white CSH powder. The CSH powder was then dry‐blended with yellow TA powder at the same organic–inorganic ratio used for the CSH‐TA copolymer, and mixed for 10 min to ensure homogeneity. Both the CSH powder and the CSH+TA mixture were hot‐pressed into bulk specimens following the same protocol as for poly(CSH‐TA).

### 4.4 Characterization of CSH‐TA Copolymer

The morphology of the CSH precursors and CSH‐TA copolymer was examined using a high‐resolution transmission electron microscope (Talos F200i, Thermo Fisher Scientific, USA) operated at 200 kV. Thermal stability and mass‐loss behavior were evaluated by thermogravimetric analysis (TGA 2(SF), Mettler Toledo, Switzerland) under a nitrogen atmosphere from 30°C–800°C at 10°C min^−1^. Phase composition was analyzed by X‐ray diffraction (Bruker D8 Advance, Germany) using Cu Kα radiation (40 kV, 30 mA) over a 2θ range of 10–70° at 2°·min^−1^. Chemical bonding environments were assessed by X‐ray photoelectron spectroscopy (K‐Alpha, Thermo Fisher Scientific, USA). Raman spectra were collected using a Renishaw 2000 Raman microscope (Renishaw, UK) equipped with a 532 nm, 20 mW laser.

In addition, the thermodynamics of the interaction between TA and CSH precursors was investigated using isothermal titration calorimetry (Nano ITC, TA Instruments, USA) at 25°C. During titration, 50 µL of 22.5 mM TA in EtOH was incrementally injected (2 µL per injection, every 120 s, total 20 injections) into 300 µL of 7.5 mM CSH in EtOH under continuous stirring at 350 rpm. Background correction was applied to account for the dilution heat of TA, and data were fitted using a single‐point (Independent) binding model.

### 4.5 Characterization of Poly(CSH‐TA) Composite

Thin lamellae of the poly(CSH‐TA) bulk were prepared by focused ion beam (FIB) milling for transmission electron microscopy analysis. Before milling, the sample surface was coated with a ∼0.2 µm platinum (Pt) layer followed by a ∼0.8 µm tungsten (W) layer to protect the material during processing. The lamellae were fabricated and lifted out using a dual‐beam FEI Helios 5UC FIB system (Thermo Fisher Scientific, USA) and transferred onto copper TEM grids. HAADF‐STEM imaging and elemental analysis were performed on a FEI Talos F200X transmission electron microscope (Thermo Fisher Scientific, USA) equipped with energy‐dispersive X‐ray spectroscopy (EDS). These measurements were performed to visualize the bicontinuous network formed by the organic and inorganic phases. Multiscale porosity was analyzed using nitrogen adsorption‐desorption (NAD) (QuadraSorb SI, Quantachrome, USA) and mercury intrusion porosimetry (MIP) (AutoPore IV 9500, Micromeritics, USA). NAD pore size distributions were derived using the Barrett‐Joyner‐Halenda model from desorption branches, whereas MIP pore diameters were calculated via the Washburn equation. Chemical structures were examined by FT‐IR spectroscopy (Nicolet Summit X, Thermo Fisher Scientific, USA) in the range of 4000–500 cm^−1^, along with S 2p analysis from XPS. Solid‐state ^29^Si MAS NMR spectra (Bruker 600 MHz spectrometer, Bruker AVANCE III, Germany) were collected to evaluate the degree of silicate polymerization before and after hot‐pressing consolidation.

### 4.6 Mechanical Characterization of the Poly(CSH‐TA) Composite

Cubic (7 mm) and prismatic (25 mm × 7 mm × 7 mm) specimens were cut precisely for compression and three‐point bending tests. All specimens were finely machined to remove potential surface defects introduced during demolding. Mechanical tests were conducted on a WDW‐100E universal servo‐hydraulic testing machine. For each group, three samples were tested, and the average values were reported. Both the compressive strength test and the three‐point bending test were performed at a loading rate of 0.2 mm min^−1^. Stress‐strain curves were recorded during loading, and the energy absorption capacity was obtained by integrating the area under the compression test curve. The crack initiation and propagation behavior of the poly(CSH‐TA) composite under compressive loading was examined by backscattered electron microscopy (BSEM). For microstructural observations, all samples were vacuum‐impregnated with epoxy resin and polished to obtain a smooth surface. Nanoindentation was performed using an iNano nanoindenter (KLA Instruments, USA). The load was increased from 0 to 50 mN within 10 s and subsequently unloaded to 0 mN over another 10 s. Hardness and elastic modulus were determined from the unloading segment using the Oliver‐Pharr method. Dynamic mechanical analysis (DMA) was conducted using a TA Q800 analyzer (TA Instruments, USA) in three‐point bending mode at 1 Hz, heating at 5°C·min^−1^ across 0°C–200°C.

### 4.7 Reprocessibility and Recyclability Testing of Poly(CSH‐TA) Composite

The physical reprocessibility of the poly(CSH‐TA) composite was evaluated on specimens of the bulk material, grinding it into powder and repressing it following the procedure described in Section 4.2. Recycled powder was prepared after 1, 3, and 5 reprocessing cycles and tested for compressive strength and energy absorption capacity using the same mechanical testing protocols as the pristine samples. For each recycling group, three specimens were tested, and the median value was reported. In‐situ UV‐vis spectroscopy (Unico UV‐2800A) was used to monitor the depolymerization process. The baseline was corrected once at the start of the measurement using ethanol as a reference; thereafter, absorbance spectra (200–600 nm) were recorded every 20 min over the period of 20–120 min.

## Author Contributions

**Zonglin Xie** and **Suning Li** contributed equally to this work. **Fuwen Zhong** and **Gongkun Xiang** contributed to writing, reviewing, and editing. **Mustapha Jamaa Garba** and **Yi Tian** performed data curation. **Jun Wu** and **Zhiwu Yu** contributed to conceptualization and supervision. **Qiang Yuan** acquired funding for the research work.

## Conflicts of Interest

The authors declare no conflicts of interest.

## Supporting information




**Supporting File**: advs77041‐sup‐0001‐SuppMat.docx.

## Data Availability

The data that support the findings of this study are available from the corresponding author upon reasonable request.
