# Supplementary material for: Self‐Assembly of Organic–Inorganic Copolymers Toward Bio‐Inspired Cementitious Composites
Source: Adv Sci (Weinh). 2026 Aug 7;13(60):e77041. doi: 10.1002/advs.77041 (PMC13449540; doi:10.1002/advs.77041)
Supplement: Supplementary file 1 [file ADVS-13-e77041-s001.docx]

**Supporting Information**

Figure S1 demonstrated the colloidal characteristics of the CSH precursor sol in ethanol (EtOH). As shown in Figure S1a, at a concentration of 30 mmol L^-1^, the sol displayed a distinct Tyndall effect under illumination, confirming the existence of nanoscale colloidal particles in contrast to the pure EtOH solvent. Figure S1b presented a TEM image revealing irregular, quasi-spherical CSH nanoparticles with characteristic diameters ranging from 3 to 10 nm.

Figure S1. (a) Optical photograph of the CSH-EtOH sol and (b) TEM image of the CSH precursor.

Figure S2 demonstrated the advanced ITC measurement used to evaluate the thermodynamic interaction between CSH and TA molecules. As shown, the measured heat values (red curve) exhibited distinct exothermic peaks upon each injection of TA. Although the raw data contained the dilution heat of TA-solvent, this contribution was negligible compared with the total heat, as confirmed by the TA-solvent (blue curve). By subtracting the dilution heat, the corrected values (yellow curve) accurately represented the specific interaction between CSH and TA. Moreover, a well-defined plateau in the heat signal further confirmed the reliability of the experimental concentration and the validity of the thermodynamic fitting.

Figure S2. The heat change during the titration of TA in CSH-EtOH and pure solvent.

To further determine the optimal binding ratio between CSH and TA, ITC measurements were performed by titrating TA-EtOH of 15, 22.5, and 37.5 mM into a 7.5 mM CSH-EtOH, corresponding to TA/CSH molar ratios of 2:1, 3:1, and 5:1, respectively. (Figure S3). At a low titrant concentration (TA/CSH = 2:1), the heat values exhibited a rising trend in cumulative heat, suggesting incomplete occupation of available coordination sites. In contrast, at a higher concentration (TA/CSH = 5:1), the heat plateau appeared obviously due to rapid site saturation. Meanwhile, the exothermic enthalpy (ΔH) increased with increasing TA concentration, reflecting stronger complexation and enhanced interfacial interactions. Despite these different TA concentrations, an equilibrium near a mole ratio (TA/CSH) of ~0.3 was shown, confirming that the binding thermodynamics is an intrinsic property of the CSH-TA system.

Figure S3. Concentration effect of TA on the normalized interaction enthalpy as a function of mole ratio.

Figure S4 illustrates the preparation route for obtaining the CSH-TA copolymer powder. In Figure S4a, introducing the CSH precursor sol into the TA-EtOH solution and stirring for 8 h produced a stable, pale-yellow sol, indicative of uniform nanoscale dispersion. Subsequent vacuum filtration (Figure S4b) transformed the colloidal sol into a cohesive pale-yellow gel, confirming the successful formation of the hybrid organo-inorganic network. Finally, vacuum desiccation at room temperature for 24 h (Figure S4c) removed residual solvent and yielded a friable solid that could be readily ground into powder. This CSH-TA copolymer powder was then used for subsequent processing.

Figure S4. (a) Optical photograph of CSH sol, TA-EtOH and CSH-TA sol, (b) Optical photograph of the pale-yellow CSH-TA gel, (c) Optical photograph of the dried CSH-TA bulk.

Figure S5 presents the Si 2p XPS spectra of the CSH-TA copolymer and the CSH precursor. Both exhibited a single Si 2p peak centered at ~102.4 eV, and no detectable chemical shift was observed between the two samples. This invariance confirmed that the local electronic environment of Si remains essentially unchanged, indicating an absence of significant interaction between the silicate sites and the -COO^-^ groups of TA. Consistent with the discussion in Figure 2h, the formation of the CSH-TA copolymer was therefore governed predominantly by enthalpy-driven coordination between Ca^2+^ and the -COO^-^ groups, rather than any perturbation of the silicate framework.

Figure S5. XPS spectra of Si 2p and Ca 2p in CSH-TA copolymer and CSH precursor.

Table S1 reports the inorganic composition of the CSH-TA residue obtained after the TG test. A portion of the S element remained incorporated within the mineral matrix, and a small amount of Cl was also detected, originating from deposition during synthesis.

Taking 100 g of CSH-TA as an example, the residual mass above 500°C is 57.1 g. According to Table S1, the residue contains 39.7 wt% CaO, corresponding to:

$$\begin{aligned} \text{m}_{\text{CaO}}\text{=57.1×39.7\%=22.67}\text{ }\text{g}\#\text{(}\text{1)} \end{aligned}$$

$$\begin{aligned} \text{n}_{\text{Ca}}\text{=}\frac{\text{22.67}}{\text{56.08}}\text{=0.404}\text{ }\text{mol}\#\text{(}\text{2)} \end{aligned}$$

The organic mass loss between 300 and 500 °C is 20.6 g (shown in Figure 2e), corresponding to:

$$\begin{aligned} \text{n}_{\text{TA}}^{\text{loss}}\text{=}\frac{\text{20.6}}{\text{206.33}}\text{=0.10 mol}\#\text{(}\text{3)} \end{aligned}$$

In addition, the residue contains 12.1 wt.% SO_3_. Since sulfur was introduced exclusively from TA during synthesis, the residual SO_3_ was attributed to sulfur retained from TA after thermal oxidation. Considering that each TA molecule contains two sulfur atoms,

$$\begin{aligned} \text{n}_{\text{TA}}^{\text{residue}}\text{=}\frac{\text{57.1×12.1\%}}{\text{160.12}}\text{=0.0431 mol}\#\text{(}\text{4)} \end{aligned}$$

Therefore,

$$\begin{aligned} \text{Ca:TA}\text{=}\frac{\text{0.407}}{\text{0.098+0.0435}}\text{=2.84:1}\#\text{(}\text{5)} \end{aligned}$$

corresponding to a Ca: TA molar ratio of 2.84: 1.

In addition, the calculated Ca/Si ratio was 0.92, which was higher than the nominal Ca/Si ratio of 0.83 based on material proportions. This discrepancy was attributed to the incomplete hydrolysis of TEOS during synthesis.

Table S1. The chemical compositions of CSH-TA residue

|  | CaO | SiO_2_ | SO_3_ | Cl |
| --- | --- | --- | --- | --- |
| CSH-TA residue | 39.7% | 43.8% | 12.1% | 1.4% |

Figure S6a shows a clear contrast between organic and inorganic phases in the HAADF-STEM image at the nanoscale, with the TA-rich organic phase appearing darker and the CSH-rich inorganic phase brighter. The two phases are spatially interwoven and distributed in a tortuous and staggered manner, forming an interpenetrating organic–inorganic network. Figure S6b reveals the overall geometry of the lamella after thinning, confirming the successful preparation of a continuous region suitable for TEM observation. Figure S6c further reveals a uniform contrast across the entire lamella at the micrometer scale, with no visible phase separation, layering, or interfacial discontinuities. This uniform contrast indicates that the poly(CSH-TA) exhibits a dense and homogeneous structure at the micrometer scale.

Figure S6. (a) Elemental mapping of Si and S merged image of poly(CSH-TA), (b) FIB-SEM cross-section image, (c) Bright-field transmission electron microscopy (BF-TEM) image of FIB-prepared sample.

Figure S7 presents a schematic illustration of silicate tetrahedra in distinct coordination environments within the CSH silicate network. In this notation, a Q^n^ unit refers to a SiO_4_ tetrahedron in which n of the four oxygen atoms participate in bridging Si-O-Si linkages. Specifically, Q^1^ units correspond to end-chain (terminal) silicates, where each tetrahedron is connected to the chain through a single bridging oxygen. Q^2^ units represent bridging silicates, in which two bridging oxygens link each tetrahedron to neighbouring units, forming the characteristic dreierketten chain of CSH. Q^3^ units denote branching silicates, where three bridging oxygens extend connectivity in two dimensions, forming cross-linked or sheet-like structures.

Figure S7. Schematic illustration of silicate tetrahedra in different coordination environments, representing Q^1^ end-chain units, Q^2^ bridging units, and higher-order Q^3^ sites within the CSH silicate network.

Figure S8 provides the FTIR spectra of pure CSH powders before and after pressing treatment. The broad absorption bands in the 900-1100 cm^-1^ region are assigned to Si-O stretching vibrations of silicate tetrahedra. With increasing pressing treatment, the Si-O stretching envelope becomes broadened and slightly reshaped, indicating changes in the local silicate environments. To further analyze these variations, the 900-1100 cm^-1^ region was deconvoluted into two overlapping components, including a lower-wavenumber Q^2^-related band centred at ~957 cm^-1^ and a higher-wavenumber Q^3^-related shoulder. The Q^3^-related shoulder exhibits a slight shift toward higher wavenumbers from ~998 to ~1008 cm^-1^ after pressing, suggesting pressure-induced redistribution of silicate structural units.

Figure S8. FTIR spectra of amorphous CSH before and after pressing, together with deconvolution of the Si-O stretching region (750-1300 cm⁻^1^).

Figure S9 shows optical images of the poly(CSH-TA) composite during uniaxial compression at different strain levels. Poly(CSH-TA) composite remained geometrically stable under uniaxial compression up to 5% strain, showing no visible cracks or deformation-induced defects. At 10% strain, slight edge spalling appeared at the bottom-right corner, indicating the onset of localized damage. Nevertheless, the overall specimen preserved its integrity and continued to deform in a largely homogeneous manner, suggesting that damage initiation occurred only at higher strain levels and the composite possesses good resistance to early compressive failure.

Figure S9. Optical images of the poly(CSH-TA) composite during uniaxial compression at different strain levels (0%, 5%, and 10%).

Table S2. Comparison of compressive strength and energy absorption capacity among poly(CSH-TA), bio-minerals, and other typical engineering materials.

| **Name** | **Compressive Strength (MPa)** | **Energy Absorption Capacity (J·cm^-3^)** | **Reference** |
| --- | --- | --- | --- |
| UHPC | 160 | 0.8 | ^[1]^ |
|  | 140 | 0.84 | ^[2]^ |
|  | 220 | 0.77 | ^[2]^ |
|  | 150 | 0.6 | ^[3]^ |
|  | 243 | 0.37665 | ^[4]^ |
| Cement, Concrete | 60 | 0.111 | ^[5]^ |
|  | 48 | 0.0528 | ^[5]^ |
|  | 64 | 0.1184 | ^[6]^ |
|  | 90 | 0.108 | ^[7]^ |
|  | 88.5 | 0.092925 | ^[8]^ |
|  | 87 | 0.0783 | ^[9]^ |
| Polymer-modified Cementitious Materials | 100 | 0.315 | ^[10]^ |
|  | 43.58 | 0.712 | ^[10]^ |
|  | 65.47 | 0.596 | ^[10]^ |
|  | 25.79 | 0.73 | ^[11]^ |
|  | 58 | 0.145 | ^[12]^ |
|  | 40 | 0.6 | ^[12]^ |
|  | 50 | 0.4 | ^[12]^ |
| Soft Polymer | 4 | 1.7 | ^[13]^ |
|  | 9 | 2.25 | ^[13]^ |
|  | 10 | 4.75 | ^[14]^ |
|  | 2 | 0.9 | ^[14]^ |
|  | 7.7 | 3.60745 | ^[14]^ |
| Ceramic | 380 | 0.76 | ^[15]^ |
|  | 350 | 0.7 | ^[16]^ |
|  | 210 | 0.7875 | ^[17]^ |
|  | 700 | 0.98 | ^[17]^ |
|  | 112 | 1.232 | ^[17]^ |
| Bio-inspired Materials | 200 | 3 | ^[18]^ |
|  | 300 | 6.75 | ^[18]^ |
| Bio-minerals | 600 | 2.4 | ^[19]^ |
|  | 80 | 20 | ^[20]^ |
|  | 400 | 4 | ^[20]^ |
| poly(CSH-TA) | 312 | 15.1 | This work |

Table S3. Comparison of H^3^/E^2^ and hardness for poly(CSH-TA) composite, bio-minerals, and other typical engineering materials.

| **Name** | **Hardness *H*(GPa)** | ***H^3^/E^2^*(MPa)** | **Reference** |
| --- | --- | --- | --- |
| Bio-minerals | 3.42 | 11.239 | ^[21]^ |
|  | 0.63 | 1.55 | ^[22]^ |
|  | 0.89 | 4.236 | ^[23]^ |
|  | 3.19 | 4.541 | ^[24]^ |
|  | 0.92 | 1.968 | ^[25]^ |
|  | 3.43 | 12.244 | ^[26]^ |
| Conventional CSH | 0.43 | 0.2249519 | ^[27]^ |
|  | 0.57 | 0.2760737 | ^[27]^ |
|  | 0.66 | 0.33261141 | ^[28]^ |
|  | 0.92 | 0.55079221 | ^[28]^ |
|  | 0.98 | 0.76832 | ^[28]^ |
|  | 1.26 | 1.250235 | ^[28]^ |
| Polymer | 0.0804 | 0.1884 | ^[23]^ |
|  | 0.096 | 0.201 | ^[23]^ |
|  | 0.1104 | 0.294 | ^[29]^ |
|  | 0.1244 | 0.424 | ^[24]^ |
|  | 0.1524 | 0.2584 | ^[30]^ |
|  | 0.3484 | 0.5314 | ^[31]^ |
|  | 0.2764 | 1.979 | ^[32]^ |
|  | 0.1894 | 0.5984 | ^[33]^ |
|  | 0.0714 | 0.016 | ^[34]^ |
|  | 0.1814 | 0.7424 | ^[35]^ |
|  | 0.2384 | 1.064 | ^[36]^ |
|  | 0.2664 | 0.999 | ^[37]^ |
|  | 0.105 | 0.129 | ^[37]^ |
|  | 0.15 | 0.247 | ^[38]^ |
|  | 0.4814 | 1.497 | ^[39]^ |
|  | 0.2182 | 0.574 | ^[40]^ |
|  | 0.0072 | 0.0424 | ^[41]^ |
| Mentals, Alloys | 0.819 | 0.136 | ^[42]^ |
|  | 1.24 | 0.313 | ^[43]^ |
|  | 2.346 | 0.216 | ^[44]^ |
|  | 1.2 | 0.057 | ^[44]^ |
|  | 1.82 | 0.165 | ^[44]^ |
|  | 2.14 | 0.233 | ^[44]^ |
|  | 1.78 | 0.241 | ^[44]^ |
|  | 1.91 | 0.201 | ^[44]^ |
|  | 2.29 | 0.7644 | ^[45]^ |
|  | 1.8044 | 0.119 | ^[46]^ |
|  | 1.85 | 0.493 | ^[47]^ |
|  | 1.35 | 0.153 | ^[48]^ |
|  | 7.901 | 1.451 | ^[49]^ |
| Our work  (poly(CSH-TA)) | 2.01 | 3.78487 | This work |
| CSH | 0.67 | 1.2233 | This work |

Figure S10 presents photographs and compressive strength results of poly(CSH-TA) composites prepared under different pressing conditions. At room temperature, increasing the pressure from 10 MPa to 500 MPa led to a progressive improvement in specimen uniformity, as indicated by the gradual disappearance of visible particle boundaries in both the top and side views. Notably, a pressure of 500 MPa produced a well-densified poly(CSH-TA) monolith with high mechanical performance, and further increases in pressure led to only marginal additional strength gains. The effect of temperature became secondary once the activation threshold for TA ring-opening was reached. Pressing at 160°C under 10 MPa produced a markedly more homogeneous structure than pressing at room temperature, yet increasing the temperature further to 160°C induced minimal additional improvement in compressive strength. What’s worse, when the temperature rose to ~200°C, thermal decomposition of the CSH-TA copolymer began to occur, leading to a pronounced deterioration in mechanical performance. The compressive strength of the specimen processed at 200°C drops to only ~80 MPa.

Figure S10. Photographs and compressive strength of poly(CSH-TA) composites prepared under different pressing conditions.

Figure S11 presents the mechanical properties of pure CSH before and after repeated pressing cycles. The results show that CSH compaction is strongly affected by temperature and water state. When hot pressing was performed above 100 °C, the compacted CSH showed a strength lower than 50 MPa (Figure 4a), likely due to the partial loss of physically adsorbed and weakly bound water. In contrast, room-temperature pressing at 500 MPa enabled pure CSH to reach a compressive strength close to 200 MPa, supporting that CSH can form pressure-assisted interparticle contact bonding. Moreover, the strength remained above 100 MPa after five repeated pressing cycles, further indicating that CSH-based contact bonding can be reformed under pressure. Nevertheless, its strength retention was lower than that of poly(CSH-TA), indicating that structural reconstruction of CSH is only partially recoverable and that dynamic S-S exchange in the TA network further improves the reprocessibility of poly(CSH-TA).

Figure S11. Mechanical properties of pure CSH before and after repeated pressing cycles.

Figure S12 provides the morphology of poly(CSH-TA) samples exposed to aggressive environments, such as NaCl and Na_2_SO_4_ solutions. Across all immersion conditions and even up to 28 days, the powder samples retained robust interfaces between organic and inorganic phases, as evidenced by the absence of any yellow colouration in the supernatant, indicating negligible TA leaching. However, compared with the samples immersed in de-ionized water, the supernatants of the NaCl and Na_2_SO_4_ solutions, which represent extremely high salt concentrations of 1 mol·L^-1^, exhibit slight turbidity that was likely associated with minor calcium dissolution from the CSH phase. As a control, CSH powder after pressing immersed under the same conditions showed much stronger turbidity in the supernatant. This comparison suggests that TA coordination helped suppress, although not completely prevent, the aqueous destabilization of CSH in highly concentrated salt solutions.

Figure S12. Poly (CSH-TA) samples immersed for 28 days in three different aqueous environments.

Figure S13 presents the corresponding results for pure CSH powder under the same immersion conditions as Figure S12 for 7 days. Compared with poly(CSH-TA), the pure CSH suspensions exhibited markedly stronger turbidity under all immersion conditions, indicating poorer aqueous stability. The increased turbidity in the salt solutions suggests more severe colloidal destabilization, likely accompanied by greater Ca leaching. These results further demonstrate that TA coordination enhances the aqueous stability of CSH in highly concentrated salt solutions.

Figure S13. CSH samples immersed for 7 days in three different aqueous environments.

Figure S14 provides a schematic illustration of the chemical recycling process used to recover TA monomers from the poly(CSH-TA) nanocomposite. As the pH was adjusted to 13.8 using 1 mol·L^-1^ NaOH and maintained for increasing durations (30 min, 3 h, and 6 h), the poly(CSH-TA) powder gradually dissociated into a clear TA-containing solution with suspended white CSH particles. In this alkaline environment, hydroxide ions first disrupted the Ca^2+^-COO^-^ coordination at the organic-inorganic interface, releasing TA segments from the CSH binding sites. Once de-coordinated, the TA network underwent base-catalyzed cleavage of its dynamic disulfide bonds, leading to de-crosslinking and complete depolymerization. Filtration separated the inorganic CSH fraction (white sample), while subsequent acidification of the filtrate protonated TA and induced its precipitation as pale-yellow solids. The precipitated TA monomers were collected by filtration, providing an efficient and solvent-minimized closed-loop route for TA recovery.

Figure S14. Chemical recycling process used to recover TA monomers from the poly(CSH-TA) composite.

**Reference**

[1]	K. Wille, A. E. Naaman, G. J. Parra-Montesinos, *ACI Materials Journal* **2011**, *108*, 46.

[2]	N. M. Azreen, R. S. M. Rashid, Y. H. Mugahed Amran, Y. L. Voo, M. Haniza, M. Hairie, R. Alyousef, H. Alabduljabbar, *Construction and Building Materials* **2020**, *263*, 120161.

[3]	S. S. Zhang, J. J. Wang, G. Lin, T. Yu, D. Fernando, *Construction and Building Materials* **2023**, *370*, 130658.

[4]	A. Tafraoui, G. Escadeillas, S. Lebaili, T. Vidal, *Construction and Building Materials* **2009**, *23*, 669.

[5]	P. Desnerck, G. De Schutter, L. Taerwe, *Structural Concrete* **2012**, *13*, 95.

[6]	B. De Nicolo, L. Pani, E. Pozzo, *Materials and Structures* **1994**, *27*, 206.

[7]	L. R. Taerwe, **n.d.**

[8]	D. C. Jansen, S. P. Shah, *Journal of Engineering Mechanics* **1997**, *123*, 25.

[9]	M. H. Zhang, O. E. Gjvorv, **n.d.**

[10]	M. Abbasnejadfard, M. Bastami, S.-A. Hashemi, *Journal of Building Engineering* **2022**, *48*, 103966.

[11]	J. Gu, J. Xing, J. Che, S. C. Chin, *Physics and Chemistry of the Earth, Parts A/B/C* **2025**, *139*, 103895.

[12]	Z. Li, A. Liu, D. Gao, C. Wu, X. Liu, H. Zhai, *Coatings* **2023**, *13*, 1964.

[13]	N. Sultana, M. Wang, *Biofabrication* **2012**, *4*, 015003.

[14]	K. Nakai, T. Yokoyama, *Journal of Solid Mechanics and Materials Engineering* **2008**, *2*, 557.

[15]	R. J. Kerans, *International Journal of Ceramic Engineering & Science* **2022**, *4*, 23.

[16]	Y. Huang, W. Huang, Q. Huang, W. Tuo, Q. Feng, *Materials* **2025**, *18*, 390.

[17]	Y. Wu, Y. Zhang, S. Zhang, X. Wang, Z. Liang, W. Hu, Z. Zhao, J. He, D. Yu, B. Xu, Z. Liu, Y. Tian, *Sci. China Mater.* **2021**, *64*, 46.

[18]	J. Chen, M. Zhao, Z. Liu, F. Wang, S. Hu, *ACS Nano* **2025**, *19*, 1732.

[19]	J. Sun, B. Bhushan, *RSC Advances* **2012**, *2*, 7617.

[20]	R. Wang, H. S. Gupta, *Annual Review of Materials Research* **2011**, *41*, 41.

[21]	J. Sun, J. Tong, *Journal of Bionic Engineering* **2007**, *4*, 11.

[22]	G. X. Ni, Y. S. Choy, W. W. Lu, A. H. W. Ngan, K. Y. Chiu, Z. Y. Li, B. Tang, K. D. K. Luk, *Biomaterials* **2006**, *27*, 1963.

[23]	M. Naffakh, C. Marco, M. A. Gómez, I. Jiménez, *J. Phys. Chem. B* **2008**, *112*, 14819.

[24]	L. Shen, W. C. Tjiu, T. Liu, *Polymer* **2005**, *46*, 11969.

[25]	E. Mahoney, A. Holt, M. Swain, N. Kilpatrick, *Journal of Dentistry* **2000**, *28*, 589.

[26]	Y.-Y. Kim, L. Ribeiro, F. Maillot, O. Ward, S. J. Eichhorn, F. C. Meldrum, *Advanced Materials* **2010**, *22*, 2082.

[27]	K. Velez, S. Maximilien, D. Damidot, G. Fantozzi, F. Sorrentino, *Cement and Concrete Research* **2001**, *31*, 555.

[28]	R. Hay, J. Li, K. Celik, *Cement and Concrete Research* **2020**, *134*, 106088.

[29]	K. D. Behler, A. Stravato, V. Mochalin, G. Korneva, G. Yushin, Y. Gogotsi, *ACS Nano* **2009**, *3*, 363.

[30]	U. Maitra, K. E. Prasad, U. Ramamurty, C. N. R. Rao, *Solid State Communications* **2009**, *149*, 1693.

[31]	M. Cadek, J. N. Coleman, V. Barron, K. Hedicke, W. J. Blau, *Appl. Phys. Lett.* **2002**, *81*, 5123.

[32]	F. Mammeri, J. Teyssandier, C. Connan, E. L. Bourhis, M. M. Chehimi, *RSC Advances* **2012**, *2*, 2462.

[33]	M. Olek, K. Kempa, S. Jurga, M. Giersig, *Langmuir* **2005**, *21*, 3146.

[34]	M. Olek, K. Kempa, M. Giersig, *International Journal of Materials Research* **2006**, *97*, 1235.

[35]	S.-C. Her, T.-Y. Shiu, *adv sci lett* **2012**, *13*, 547.

[36]	M. R. Ayatollahi, S. Doagou-Rad, S. Shadlou, *Macromolecular Materials and Engineering* **2012**, *297*, 689.

[37]	R. Saito, S.-I. Kobayashi, T. Hosoya, *Journal of Applied Polymer Science* **2005**, *97*, 1835.

[38]	J. Chen, X. Guo, Q. Tang, C. Zhuang, J. Liu, S. Wu, B. D. Beake, *Carbon* **2013**, *55*, 144.

[39]	A. Molazemhosseini, H. Tourani, M. R. Naimi-Jamal, A. Khavandi, *Polymer Testing* **2013**, *32*, 525.

[40]	A. M. Díez-Pascual, M. A. Gómez-Fatou, F. Ania, A. Flores, *J. Phys. Chem. C* **2012**, *116*, 24193.

[41]	J. K. Adewole, U. A. Al-Mubaiyedh, A. Ul-Hamid, A. A. Al-Juhani, I. A. Hussein, *The Canadian Journal of Chemical Engineering* **2012**, *90*, 1066.

[42]	H. R. A. Ram, Praveennath. G. Koppad, K. T. Kashyap, *Materials Science and Engineering: A* **2013**, *559*, 920.

[43]	S. Koch, M. D. Abad, S. Renhart, H. Antrekowitsch, P. Hosemann, *Materials Science and Engineering: A* **2015**, *644*, 218.

[44]	L. Yang, Y. Chen, J. Miller, W. J. Weber, H. Bei, Y. Zhang, *Materials Science and Engineering: A* **2022**, *856*, 143685.

[45]	F. K. Mante, G. R. Baran, B. Lucas, *Biomaterials* **1999**, *20*, 1051.

[46]	P. Zhu, Y. Zhao, S. Agarwal, J. Henry, S. J. Zinkle, *Materials & Design* **2022**, *213*, 110317.

[47]	L. Liu, Y. Zhang, J. Han, X. Wang, W. Jiang, C.-T. Liu, Z. Zhang, P. K. Liaw, *Advanced Science* **2021**, *8*, 2100870.

[48]	Y. Li, K. He, C. Liao, C. Pan, *Journal of Materials Research* **2012**, *27*, 192.

[49]	W. Fang, Z. Mu, Y. He, K. Kong, K. Jiang, R. Tang, Z. Liu, *Nature* **2023**, *619*, 293.
